# Predicting drug–drug interactions through drug structural similarities and interaction networks incorporating pharmacokinetics and pharmacodynamics knowledge

**DOI:** 10.1186/s13321-017-0200-8

**Published:** 2017-03-07

**Authors:** Takako Takeda, Ming Hao, Tiejun Cheng, Stephen H. Bryant, Yanli Wang

**Affiliations:** National Center for Biotechnology Information, National Library of Medicine, National Institutes of Health, Bethesda, MD 20894 USA

**Keywords:** Drug–drug interaction (DDI), Structural similarity, Interaction networks, Enzymes, Transporters, Target proteins, Pharmacokinetics (PK), Pharmacodynamics (PD), Protein–protein interaction (PPI)

## Abstract

Drug–drug interactions (DDIs) may lead to adverse effects and potentially result in drug withdrawal from the market. Predicting DDIs during drug development would help reduce development costs and time by rigorous evaluation of drug candidates. The primary mechanisms of DDIs are based on pharmacokinetics (PK) and pharmacodynamics (PD). This study examines the effects of 2D structural similarities of drugs on DDI prediction through interaction networks including both PD and PK knowledge. Our assumption was that a query drug (Dq) and a drug to be examined (De) likely have DDI if the drugs in the interaction network of De are structurally similar to Dq. A network of De describes the associations between the drugs and the proteins relating to PK and PD for De. These include target proteins, proteins interacting with target proteins, enzymes, and transporters for De. We constructed logistic regression models for DDI prediction using only 2D structural similarities between each Dq and the drugs in the network of De. The results indicated that our models could effectively predict DDIs. It was found that integrating structural similarity scores of the drugs relating to both PK and PD of De was crucial for model performance. In particular, the combination of the target- and enzyme-related scores provided the largest increase of the predictive power.

**Electronic supplementary material:**

The online version of this article (doi:10.1186/s13321-017-0200-8) contains supplementary material, which is available to authorized users.

**Graphical abstract:**
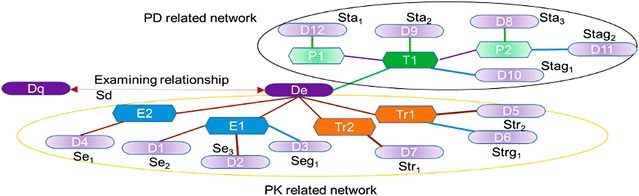
.

## Background

DDI occurs when a drug affects the efficacy of another drug that is co-administered. Between 2009 and 2012, 38.1% of U.S. adults ages 18–44 used three or more prescription drugs during a 30 day time period [1]. The percentages increased substantially as a function of age, with 67.2% for ages 45–64, and 89.8% for age 65 years or older, respectively. The number of incidents of adverse drug reaction increases exponentially if a patient takes four or more drugs [2]. Although DDI may have beneficial effects, it can cause serious adverse effects and sometimes lead to drug withdrawal [3]. During drug development, the prediction of such DDI would help reduce the time and costs by prioritizing drug candidates.

The main types of DDI are based on pharmacokinetics (PK) and pharmacodynamics (PD). PK is the body’s response to a drug, which includes absorption, distribution, metabolism, and excretion (ADME). DDI occurs when two drugs share the same mechanism of excretion [4]. A significant number of studies on PK-based DDI have been done at the molecular level involving enzymes and transporters, and resulted in a large amount of experimental data [5]. For example, changes in gastric pH caused by a drug can affect the gastro-intestinal absorption of a co-administered drug [4]. If two drugs both binding to a same plasma protein are co-administered, the concentration of the free drugs in plasma may change [4]. Also, various drugs are substrates, inhibitors, or inducers of the CYP enzymes, the dominant metabolic enzymes. As a result, DDI can occur when an inhibitor and a substrate of a CYP enzyme are co-administered. The PD-based DDIs are found at the receptor level, the signal transduction level, and the physiological system level [6]. The most common ones occur at the receptor level where drugs compete for binding to the same receptor.

Many studies for predicting DDI have been reported based on various approaches such as physiologically based pharmacokinetic (PBPK) modeling, molecular structural similarity analysis, ontology and annotation based analysis, network modeling, QSAR modeling, and data mining from clinical data. A PBPK model consists of mathematical equations that describe the properties of ADME in the human body. For example, a PBPK model was developed using the results from a clinical pharmacokinetic study under single and multiple-dose conditions to predict the DDI for crizotinib with ketoconazole or rifampin [7]. Structural similarity for DDI prediction has been employed based on the idea that if there is a DDI between drug A and drug B, and drug C has a similar structure to drug A, there is likely a DDI between drug C and drug B [8]. Vilar et al. predicted DDIs with a matrix transformation approach using structural similarities of drugs with molecular fingerprints [8]. In subsequent studies, the authors reported prediction methods using integrated similarity measures including interaction profile similarities, adverse effect similarities, and target similarities [9]. Based on the similar idea, INferring drug interactions (INDI), was developed to predict CYP-related and PD-related DDI using drug chemical similarities, side effects similarities, ATC (Anatomical Therapeutic Chemical classification system) similarities, target sequence similarities, protein–protein interaction similarities, and Gene Ontology similarities [10]. In addition, 3D pharmacophoric similarity was used for the prediction of DDI, and the significance of 3D structure data was demonstrated, which captured the characteristics that were missed by using only 2D data [11]. Luo et al. developed a web server for DDI prediction through chemical–protein interaction profiles created by docking chemicals to the ligand binding pockets of the collected PDB structures [12]. DDI prediction using machine learning approaches was implemented on DDI-networks through integrated phenotypic, therapeutic, structural and genomic similarities [13]. QSAR models for DDI prediction were constructed for CYP1A2, 2C9, 2D6, and 3A4 by using two types of chemical descriptors and the balanced accuracy ranged from 72 to 79% [14]. There are also knowledge-based studies for DDI prediction. Herrero-Zazo et al. inferred DDI with DDI knowledge including types, mechanisms, and applications of DDIs using semantic web rule language [15]. Huang et al. predicted DDI using protein–protein interaction network, which demonstrated an accuracy of 0.82 and recall of 0.62 [16]. Cami et al. [17] predicted DDI using known DDI networks. Recently, a computational model for predicting DDI was developed through integrated clinical side effect information from the drug labels and FDA adverse event reporting system [18]. Electronic health records (EHRs) were also used to identify or prioritize drug–drug-adverse events [19, 20].

Here, we proposed models for predicting DDIs using the structural similarities of drugs from the PK and PD networks and investigated the factors influencing DDIs for further improvement of the predictions. Our assumption is that a query drug (Dq) and a drug to be examined (De) tend to interact if Dq is structurally similar to the drugs in De’s network that interact with the enzymes/transporters/target proteins of De. The results of model assessment and two case studies were reported.

## Results and discussion

### Characteristics of each score type in the network

**Fig. 1:**
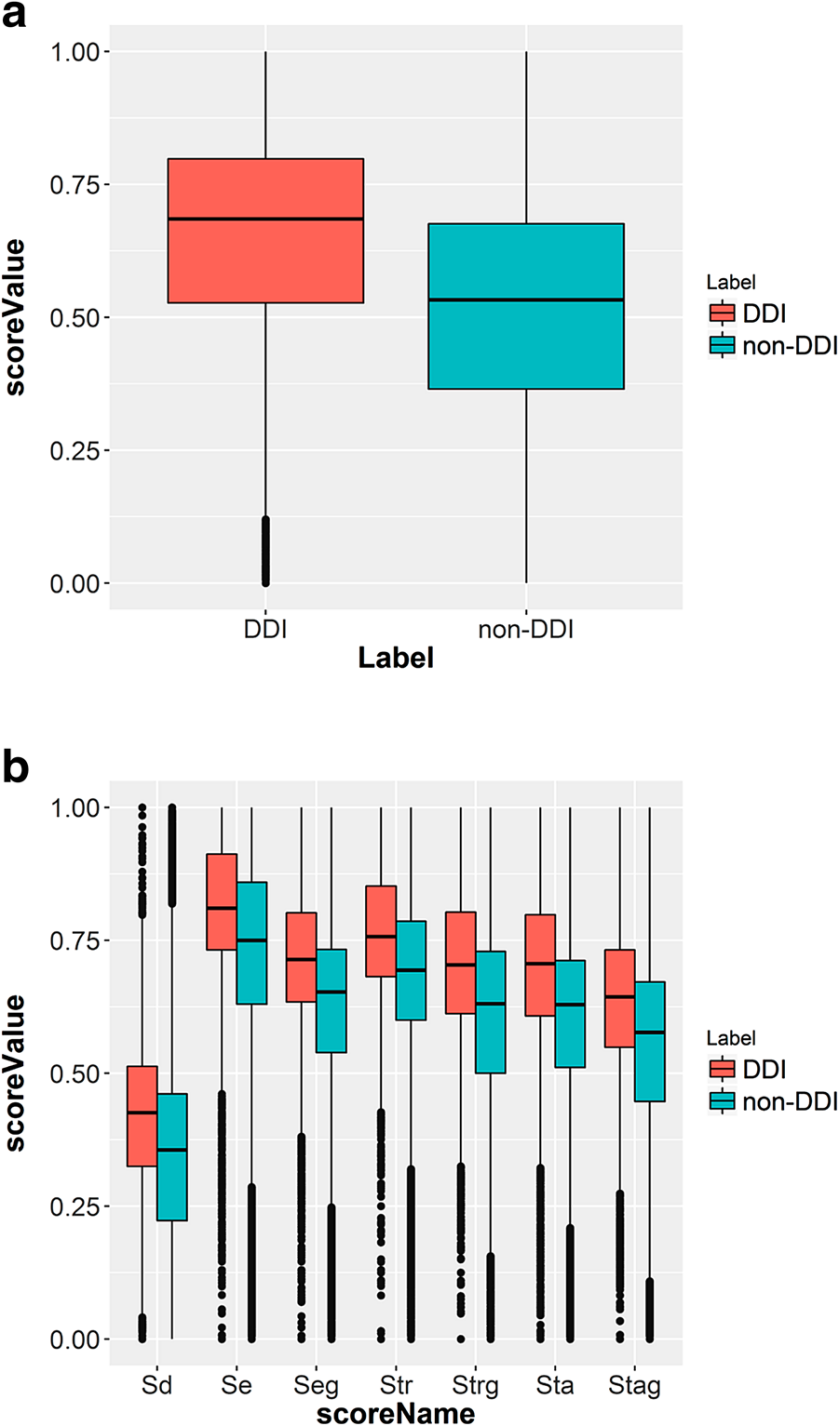
Structural similarity score distributions. **a** All types of scores combined. **b** Each individual score type

The distributions of structural similarities between Dq and the drugs in a network of De for the DDI pairs and non-DDI pairs are shown in Fig. 1. The construction of the network of De and the score types (S_d_, S_e_, S_eg_, S_tr_, S_trg_, S_ta_, and S_tag_) are described and defined in the Methods section. Note that the score values of −10 were excluded from Fig. 1. Panel (A) shows that the median of similarities for all score types together for DDI pairs is larger than that for non-DDI pairs in general. The interquartile range for DDI pairs is slightly narrower than that for non-DDI pairs. The same trends were observed in score distributions for each individual score type [panel (B)]. This suggests that the structural similarity scores based on integrated PK and PD interaction network can be used for the prediction of DDIs. The distributions of the scores S_e_, S_tr_, and S_ta_ shifted to the higher value range comparing to those of the corresponding scores for pharmacogenetic associations (S_eg_, S_trg_, and S_tag_), which implies that pharmacogenetic interactions are less structurally dependent than physical interactions.

The averages of S_d_ in both cases of DDIs and non-DDIs (0.416 and 0.346 respectively) were the lowest among the scores, while the other scores (S_e_, S_eg_, S_tr_, S_trg_, S_ta_, and S_tag_) ranged from 0.556 to 0.800 (Additional file 1: Table S1). Even though the structure of Dq is dissimilar with De, the other drugs interacting with the enzymes, transporters, or targets in De’s network can be structurally similar to Dq. In this case, it is still possible that Dq interacts with those proteins of De and DDI between Dq and De may be observed. S_e_ showed the highest average score value, which may be explained by the fact that an enzyme can metabolize many drugs, and therefore the probability for finding drugs structurally similar to Dq in the network can be higher.

**Fig. 2:**
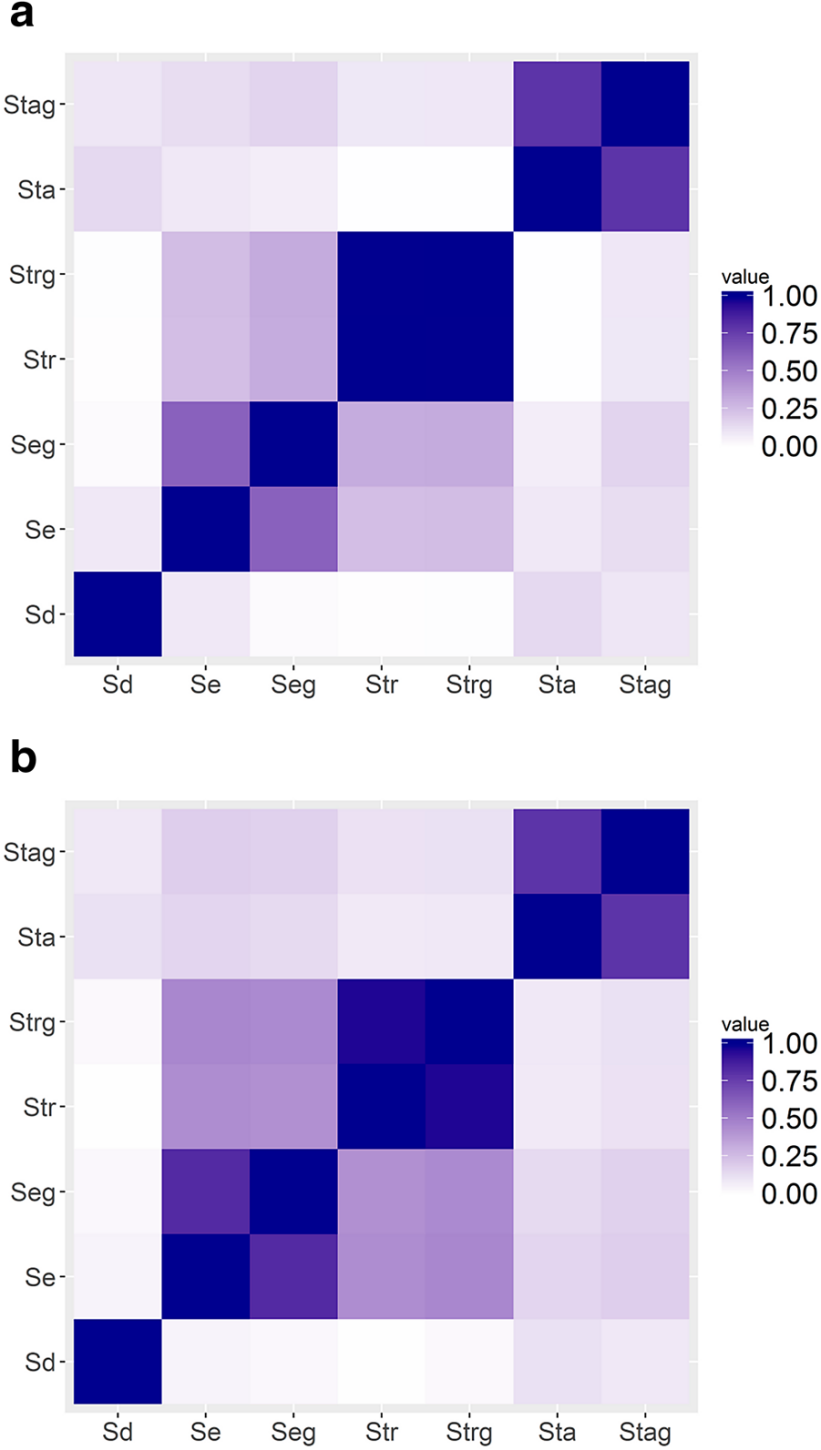
Correlation between scores. **a** DDI, **b** non-DDI

The correlations between scores for DDI and non-DDI pairs are shown in Fig. 2. It appears that correlations among the scores are generally classified into three groups: S_d_, (S_e_, S_eg_, S_tr_, and S_trg_), and (S_ta_ and S_tag_), for both DDI and non-DDI pairs. Enzyme (S_e_ and S_eg_) and transporter (S_tr_ and S_trg_) related scores correlated with each other to some degree, which may be related to the interplay between metabolizing enzymes and transporters. It is reported that metabolizing enzymes and transporters influence each other for the ADME of drugs and therefore may affect DDI [21]. For example, many drugs metabolized by CYP3A4 are also transported by P-glycoprotein [22]. Also, physical interaction and pharmacogenetic association correlated strongly, i.e. (S_e_ and S_eg_), (S_tr_ and S_trg_), and (S_ta_ and S_tag_). However, the correlations among the scores for DDI pairs are slightly weaker than those for non-DDI pairs.

### Prediction results

Average area under the curve (AUC) values for the 4-fold cross-validations with a series of score combination schemes are shown in Table 1. Generally, combining similarity scores that include both information relating to PK and PD resulted in stronger predictions. Although AUCs of the regression models using Set 1 through Set 6 were not significantly different with the average values in the range of 0.84 and 0.83, ANOVA test revealed the importance of considering multiple scores. This implies the merit of information integration to our DDI prediction model using the interaction network. Results for Set 8 and Set 9, both integrating PK information regarding transporters and PD information (PKtr + PD), showed lower AUC than those for Set 1 through Set 6 which all included enzyme information.

**Table 1:** AUC for 4-fold cross-validations

Score set	Scores	Included information	Average AUC	SD
Set 1	S_d_, S_e_, S_eg_, S_tr_, S_trg_, S_ta_, S_tag_	DR + PK + PD	0.837	0.005
Set 2	S_e_, S_tr_, S_ta_	(PK + PD)_nog	0.837	0.009
Set 3	S_e_, S_eg_, S_tr_, S_trg_, S_ta_, S_tag_	PK + PD	0.834	0.012
Set 4	S_d_, S_e_, S_tr_, S_ta_	DR + (PK + PD)_nog	0.834	0.005
Set 5	S_d_, S_e_, S_eg_, S_ta_, S_tag_	DR + PKe + PD	0.828	0.006
Set 6	S_e_, S_eg_, S_ta_, S_tag_	PKe + PD	0.827	0.008
Set 7	max(S_d_, S_e_, S_eg_, S_tr_, S_trg_, S_ta_, S_tag_)	Maximum score in the whole network	0.786	0.012
Set 8	S_tr_, S_trg_, S_ta_, S_tag_	PKtr + PD	0.741	0.009
Set 9	S_d_, S_tr_, S_trg_, S_ta_, S_tag_	DR + PKtr + PD	0.736	0.005
Set 10	S_d_, S_e_, S_eg_, S_tr_, S_trg_	DR + PK	0.672	0.006
Set 11	S_e_, S_eg_, S_tr_, S_trg_	PK	0.657	0.007
Set 12	S_d_, S_tr_, S_trg_	DR + PKtr	0.653	0.008
Set 13	S_tr_, S_trg_	PKtr	0.631	0.019
Set 14	S_ta_, S_tag_	PD	0.627	0.005
Set 15	S_ta_	PD_nog	0.620	0.008
Set 16	S_d_, S_e_, S_eg_	DR + PKe	0.619	0.002
Set 17	S_d_, S_ta_, S_tag_	DR + PD	0.617	0.003
Set 18	S_tr_	PKtr_nog	0.616	0.015
Set 19	S_d_	DR	0.601	0.007
Set 20	S_e_, S_eg_	PKe	0.593	0.009
Set 21	S_e_	Pke_nog	0.587	0.009

(PK + PD)_nog, PK and PD information without genetic information; DR, direct similarity score; PKe, PK with only enzyme information; PKtr, PK with only transporter information; SD, standard deviation

**Fig. 3:**
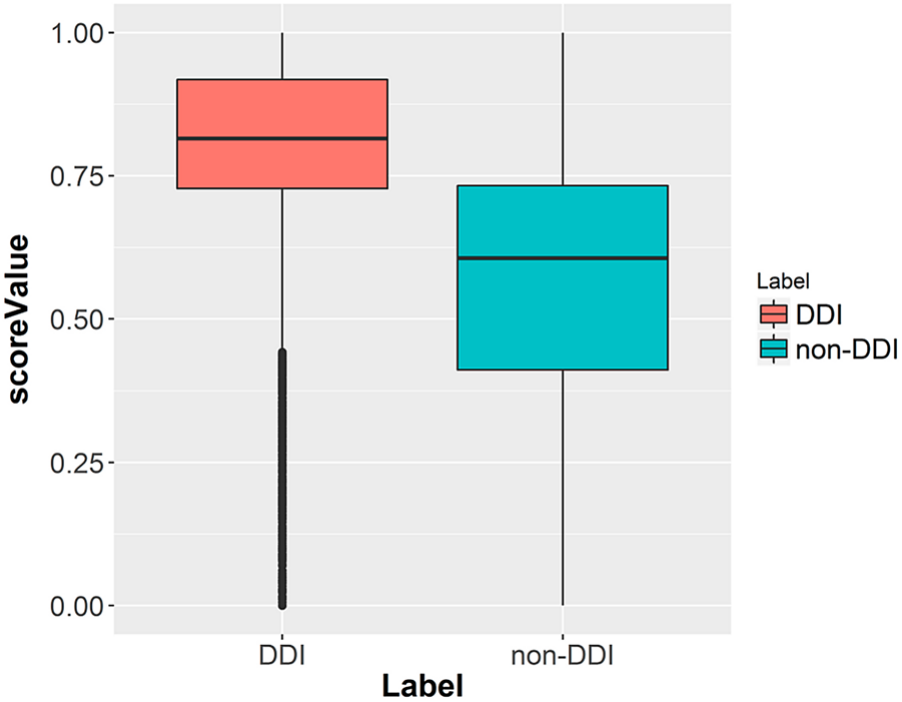
Similarity score distribution for the maximum score in the whole network

Interestingly, using a maximum score among scores in the entire network (Set 7) resulted in an AUC of 0.786 with a standard deviation of 0.012, which was close to the AUCs for the models using Set 1 through Set 6. As shown in Fig. 3, the interquartile ranges of the distributions for the maximum score in the whole network for DDI pairs and for non-DDI pairs hardly overlap, unlike the situation when all scores were considered as shown in Fig. 1a. These observations imply that the most structurally similar drug to Dq in the network is quite important to DDI prediction but it is not the decisive factor for the ultimate prediction in the network system as the AUC for this model is still smaller than that for the model using Set 1. Using only a single information type (enzymes, transporters, or targets) along with knowledge of the corresponding pharmacogenetic association resulted in lower prediction performance with AUC values ranging from 0.587 to 0.613 for the results of Set 13, Set 14, and Set 20.

There was a large increase in AUC for Set 1 through Set 6 when the PD-related information was integrated to the enzyme information (Set 20). AUC boosted from 0.593 (Set 20) to 0.827 (Set 6) with a 39% increase and from 0.627 (Set 14) to 0.827 (Set 6) with a 32% increase. The second largest improvement in AUC was observed when integrating PK-related information with PD-related information. AUC improved from 0.627 (Set 14) to 0.834 (Set 3) with a 33% increase. When adding PD-related information to the enzyme and transporter information (score Set 11), AUC jumped from 0.657 (Set 11) to 0.834 (Set 3) with a 27% increase. These results suggested that both PK and PD of De are critical for DDI prediction.

When combined with the target information, the enzyme information contributed more to the prediction than the transporter information. When the transporter data (PKtr) were replaced by the enzymes data (PKe), AUC increased from 0.741 (Set 8) to 0.827 (Set 6) with an 11% change. The structural space of drugs covered by Set 6 (PKe + PD information) might be larger than that covered by Set 8 (PKtr + PD information), which may be attributed to the fact that the number of drugs with similar structures to Dq from the enzyme-related sub-network of De is more than that from the transporter-related sub-network. Prediction performance might be improved when the number of drugs associated with transporters increases. These results could also be due to the fact that the correlation between S_e_ and S_eg_ was lower than that between S_tr_ and S_trg_ do (Fig. 2).

Comparing the results using score (Set 2 and Set 3), (Set 20 and Set 21), (Set 13 and Set 18), and (Set 14 and Set 15) revealed that pharmacogenetic associations did not contribute much to DDI prediction in terms of AUC, although the ANOVA test result indicated the importance of integrating pharmacogenetic associated information to models. This observation might be due to the fact that the scores for S_e_, S_tr_ and S_ta_ are higher in general than the corresponding scores for S_eg_, S_trg_ and S_tag_ based on the distribution shown in Fig. 1 b. Also S_e_ and S_eg_, S_tr_ and S_trg_, and S_ta_ and S_tag_ correlated with each other in both DDI and non-DDI cases to some degree (Fig. 2), again indicating that these scores have less effect on DDI prediction.

### Case studies

Two case studies of DDIs predictions are presented for warfarin and simvastatin. Warfarin is a blood thinner drug. One of warfarin’s drawbacks is that it interacts with many medications that are co-administered. Simvastatin is a drug for lowering the level of low-density lipoprotein cholesterol and fats, and for raising the level of high-density lipoprotein cholesterol in the blood. It is on the WHO model list of essential medicines [23]. For each case study we re-built models using the entire dataset but leaving out the data for any warfarin-drug pairs, or the data for any simvastatin-drug pairs, respectively, instead of applying the models constructed during the 4-fold cross validations. The model constructed with Set 1 was used for the prediction based on its superior performance according to the ANOVA test results.

#### Warfarin

The top ten drugs with predicted DDI for warfarin are listed in the Additional file 1: Table S2-1. Four are reported in DrugBank to have DDI indications. Newly predicted DDI candidates for warfarin were dronabiol, quercetin, genistein, salicylic acid, fluorescein, and doxepin. Among them, the DDI between doxepin and warfarin is reported on Micromedex [24] with a moderate interaction that increases the risk of bleeding. Quercetin is reported in Drugs.com as having moderate interactions with warfarin that reduce the efficacy of warfarin [25]. The definition of “moderate” on Drugs.com is that the combination is moderately significant in clinical applications and usually the combination should be avoided or may be used only under special circumstances. Genistein is listed as having significant interaction with warfarin on rxlist.com. The definition of “significant” interaction in rxlist.com is that the combination potentially can cause dangerous DDI and should be used with cautions and close monitoring. It is reported that quercetin displaces warfarin bound to human serum albumin [26] due to competitive binding and that genistein also shares the binding sites in human serum albumin with warfarin [27]. It is reported that special precautions are necessary when taking dronabinol together with warfarin [28]. Overall, DDI between warfarin and eight out of the top ten predicted drugs were supported by reports in literature and databases. Comparing to the prediction results in the study by Vilar et al. [8], which also used drug structural similarities, two of the top ten drugs predicted by our model (i.e. salicyclic acid and estrone) were predicted to have DDI with warfarin in their study. Their predictions are based on the structural similarity between De and the drugs that are known to have DDI with warfarin (Dq) and therefore the chemical space searched is limited. On the other hand, our approach is based on the structural similarity between Dq and the drugs that interact with the proteins in the interaction network of De. Therefore, our approach explores a larger chemical space and is capable of picking up DDIs with the drugs, which may not be structurally similar to drugs having known DDIs with warfarin.

#### Simvastatin

The top ten drugs with predicted DDI for simvastatin are shown in the Additional file 1: Table S2-2. None of the top ten drugs in Additional file 1: Table S2-2 is reported in DrugBank. Among them, however, lovastatin, prednisolone, dexamethasone, prednisone, and tacrolimus are listed by Drugs.com as having moderate interaction with simvastatin [29–33]. It is not surprising to see structurally similar drugs to simvastatin, e.g. lovastatin. However, our model also predicted tacrolimus, whose structure is not similar to simvastatin. A study reported that lovastatin and simvastatin likely had DDIs through p-glycoprotein (MDR1) transporter [34]. Six out of the top ten drugs were steroid hormones: several from the glucocorticoid family (prednisolone, dexamethasone, and prednisone), testosterone, aldosterone, and norethisterone. Dehydroepiandrosterone sulfate is the metabolite of a steroid hormone, dehydroepiandrosterone. There is a recent report that simvastatin influenced the steroid hormone level in plasma in female patients who had non-classic congenital adrenal hyperplasm and were taking metformin [35]. Overall, five out of the top ten predicted drugs were supported by reports in the literature and databases. No false negative prediction of DDIs for simvastatin was made. All known drugs having DDI with simvastatin which include a total of 31 from DrugBank were picked up by our model. In comparison, only four of our top ten drugs (i.e. testosterone, prednisolone, prednisone, and lovastatin) were predicted to have DDI with simvastatin in the study by Vilar et al. [8].

These two case studies suggested that our approach could also be used to predict possible enzymes and transporters for a drug (Dq) in general. Cytochrome P450 2C9 metabolizes warfarin and seven out of the top ten drugs predicted having DDI with warfarin. Similarly, multidrug resistance protein 1 interacts with warfarin and transports eight out of the top ten drugs. Cytochrome P450 3A4 metabolizes simvastatin and seven out of the top ten drugs in the DDI prediction, and multidrug resistance protein 1 transports simvastatin and eight out of the top ten drugs. On the other hand, the warfarin case study suggested the limitation of this approach. Comparing to that no false negative prediction of DDIs was made for simvastatin, the DDI prediction for warfarin resulted in 18 false negatives out of 150 known DDIs. This is possibly due to the lack of relevant enzyme- and transporter-information for those drugs in the network. This limitation may be eliminated over time when additional experimental PK data becomes available.

## Conclusions

In this study, we investigated the factors for predicting DDI through structural similarities and the interaction networks which contain PK and PD knowledge. Our work demonstrated: (1) structural similarities between Dq and the drugs in the network of De can be used for predicting DDIs between Dq and De; (2) the integration of both structural similarity scores relating to PK and PD was crucial for DDI prediction; (3) the inclusion of pharmacogenetically associated knowledge (scores: S_eg_, S_trg_, and S_tag_) only made minor contribution to DDI predictions. Two case studies showed the ability of this approach for predicting DDI. Eight out of the top ten predicted DDIs with warfarin, and five out of the top ten predicted DDIs with simvastatin were supported by reports in literature and multiple databases.

A limitation for the current prediction method is that it requires enzyme or transporter information for De. Imputing enzymes or transporters for the drug may be a possible solution for future study. Another limitation lies in the fact that it can only apply to small molecule drugs (i.e. not to peptides or nucleic acids). For further improving prediction, integrating other knowledge may be one direction. For example, the population of the transporter protein may depend on the cell type and intracellular membranes type [36], and therefore, tissue specific population data of transporters might help further distinguish DDI from non-DDI pairs. For enzymes, the information of the drugs such as the inducer, inhibitor, or substrate information might help enhance DDI prediction as well. Furthermore, associating the information of absorption, signal transduction pathway, physiological agonism/antagonism, or excretion (e.g. half-life) might help improve prediction performance and understand the mechanism of DDIs.

## Methods

Drug–drug interactions and association data of drug-enzyme, drug-transporter and drug-target were retrieved from DrugBank version 4.1 (downloaded on Sep. 8, 2014) [37]. This includes, 4002 drugs with fingerprints (drug set M), out of which 925 have drug–drug interaction annotations (drug set N, Additional file 1: Text S1) corresponding to 24,149 drug–drug interactions, 3448 drug-enzyme pairs, 1759 drug-transporter pairs, and 15,771 drug–target pairs. We treated the drug pairs without drug–drug interaction reported in DrugBank as non-DDI pairs. Drug-gene association data (pharmacogenetic data) were retrieved from PharmGKB [38] (3262 associations, downloaded on Sep. 26, 2014). Protein–protein physical interaction data was retrieved from BioGRID [39] (168,956 interactions from BIOGRID-ORGANISM-Homo_sapiens-3.2.117, downloaded on Oct. 21, 2014).

The modeling process contains four steps. First, interaction network for each De was constructed; second, the structural similarities between Dq and all the drugs in the network of De including De were computed; third, DDI prediction models were constructed using the structural similarities with logistic regression approach; finally, 4-fold cross-validation was carried for model evaluation.

**Fig. 4:**
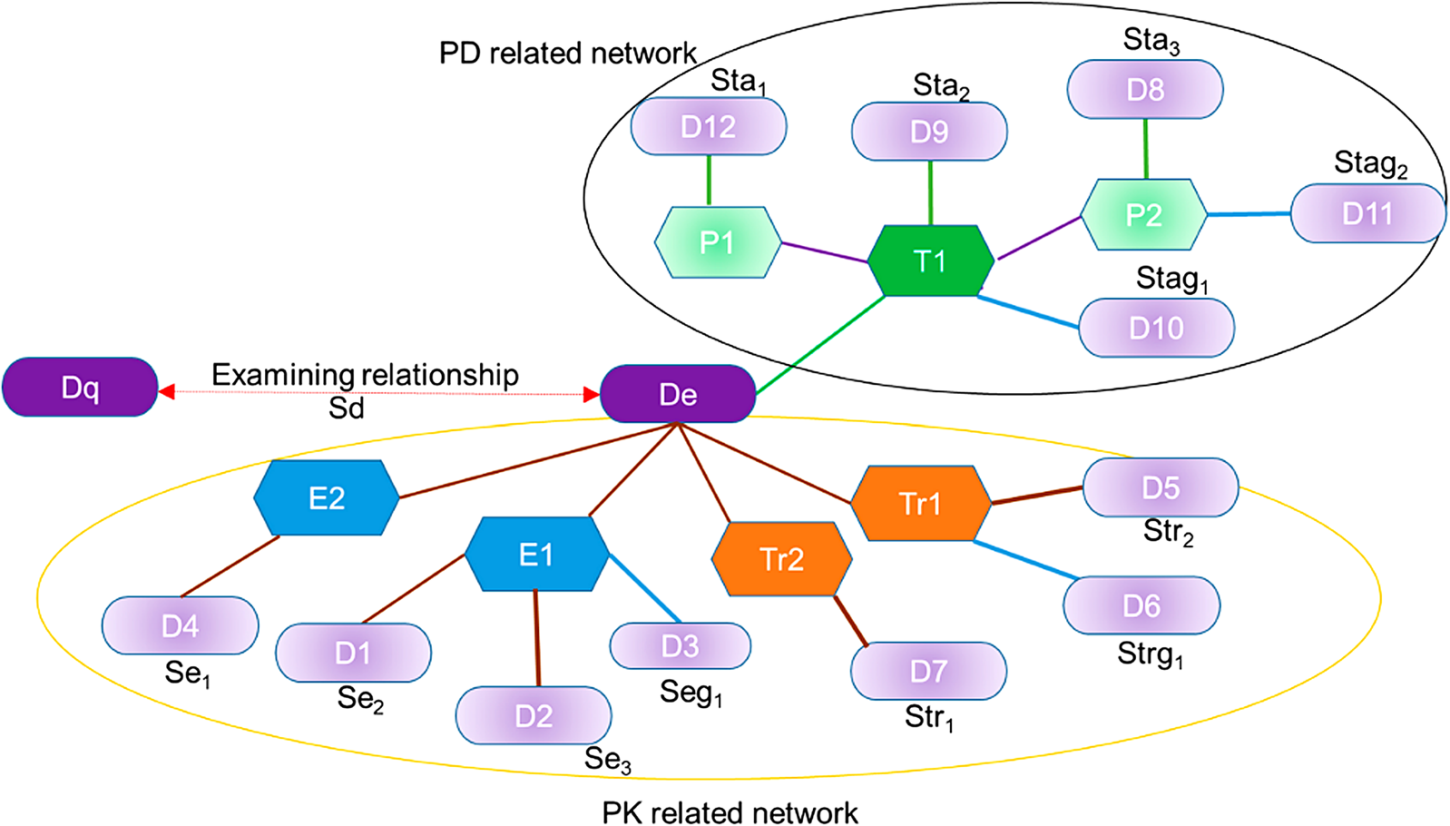
Example of a network examining relationship between two drugs (Dq and De). Dq: a query drug, for which potential DDIs are predicted with a drug under examination, De; T1: a target protein for De (*source* DrugBank); P1, P2: proteins that have physical interactions with T1 (*source* BioGRID); E1, E2: enzymes of De (*source* DrugBank); Tr1, Tr2: transporters of De (*source* DrugBank); D1 through D12: drugs associating with the proteins including T1, P1, P2, E1, E2, Tr1, and Tr2 in the network; protein–protein interaction (*source* BioGRID): *purple line*; pharmacogenetic association (*source* PharmGKB): *blue line*; PK-related interaction (*source* DrugBank): *brown line*; drug-target interaction (*source* DrugBank): *green line*. S_d_, S_e1_, S_e2_, S_e3_, S_eg1_, S_tr1_, S_tr2_, S_trg1_, S_ta1_, S_ta2_, S_ta3_, S_tag1_, S_tag2_: similarity scores between Dq and drugs in De’s network (D1 through D12)

Figure 4 illustrates a network of De which consists of two sub-networks that represent simplified PK and PD information (circled by orange and black line in Fig. 4, respectively). Short terms for describing the respective PK and PD protein types and associated drugs are provided in Fig. 4, and are used throughout the manuscript. Sub-network system presenting PD relationship was previously used by Hansen et al. [40]. Here, our assumption is that Dq and De tend to have interactions if the structure of Dq is similar to the structures of the drugs in De’s interaction network (from D1 through D12).

PK-related sub-network represents relationships between: De and the related enzymes (E1 and E2); De and its transporters (Tr1 and Tr2); E1, E2 and the drugs that interact with them (D1, D2, and D4); E1, E2 and the drugs that have pharmacogenetic associations with them (D3); Tr1, Tr2 and the drugs that they transport (D5 and D7); Tr1, Tr2 and the drugs that have pharmacogenetically related interactions with them (D6). PD-related sub-network of De represents relationships between: De and its target proteins (T1 in Fig. 4); T1 and other drugs that also target T1 (D9); T1 and the drugs that have pharmacogenetic association with T1 (D10); T1 and the proteins that physically interact with T1 (P1 and P2); P1 and P2 and the drugs that target them (D8 and D12); and P1 and P2 and the drugs that have pharmacogenetic associations with them (D11).

Our approach requires only structural similarities as the input to predict DDIs. In this work, we used PubChem 2D fingerprint [41] and Tanimoto coefficient to calculate structural similarities. Seven structural similarity scores (i.e. S_d_, S_e_, S_eg_, S_tr_, S_trg_, S_ta_, and S_tag_ as defined below) using different drug subset in De’s network were used to build DDI prediction models with logistic regression approach. Independent variables for the regression models were the scores, and the values for the dependent variable were 1 for DDI pairs and 0 for non-DDI pairs, respectively.

### Score type definitions

- S_d_: the similarity score between Dq and De.
- S_e_: the maximum similarity score between Dq and the drugs in the network of De that interact with the enzymes of De (D1, D2, and D4). S_e_ = max (S_e1_, S_e2_, S_e3_) in Fig. 4.
- S_eg_: the maximum similarity score between Dq and the drugs in the network of De that have pharmacogenetic associations with the genes of the enzymes of De (D3). S_eg_ = S_eg1_ in Fig. 4.
- S_tr_: the maximum similarity score between Dq and the drugs in the network of De that are transported by the same transporters of De (D5 and D7). S_tr_ = max (S_tr1_, S_tr2_) in Fig. 4.
- S_trg_: the maximum similarity score between Dq and the drugs in the network of De that have pharmacogenetic associations with the genes of the transporters of De (D6). S_trg_ = S_trg1_ in Fig. 4.
- S_ta_: the maximum similarity score between Dq and the drugs in the network of De that have physical interactions with the target proteins of De (D9), or the proteins that have physical interactions with the targets (D8, D12). S_ta_ = max (S_ta1_, S_ta2_, S_ta3_) in Fig. 4.
- S_tag_: the maximum similarity score between Dq and the drugs in the network of De that have pharmacogenetic associations with the genes of the target proteins of De (D10) or the proteins that have physical interactions with the targets (D11). S_tag_ = max (S_tag1_, S_tag2_) in Fig. 4.

Structural similarities were calculated for each drug in set N against the drugs in set M. If there was no drug for a category in S_e_, S_eg_, S_tr_, S_trg_ S_ta_, or S_tag_, a score −10 was assigned, which was empirically chosen for the convenience of handling scores. In all networks, Dq and De were removed when they also appeared as the drugs in the network of De. The reason we used maximum scores when there are multiple drugs in a sub-network is based on the idea that structurally similar drugs likely interact with the same protein and most structurally similar drugs probably most effectively interact with the protein and therefore have most influence on the DDI. To assess the regression models, 4-fold cross-validation was carried out for each score set (Table 1). To construct the training and test sets, drugs with no DDIs for each Dq were randomly chosen to achieve a ratio of 1:1 for the number of the DDI pairs over that of the non-DDI pairs for each Dq. Since we chose drugs from a pool of a large number of drugs with non-DDI for Dq to construct a balanced classification (DDI pairs:non-DDI pairs = 1:1), we were concerned about the bias brought by the selection of the non-DDI drugs. To examine this, we chose non-DDI drugs randomly while fixing the number of DDI drugs, built models, evaluated, and repeated 10 times. The standard deviations of the AUC ranged from 0.000 to 0.019 suggesting that the models were stable, i.e. the selection of non-DDI drugs did not affect the results. If De was the only drug in its network, it was not included in any data set. The numbers of DDI and non-DDI pairs for each score set in each test set were provided in Additional file 1: Table S3. The logistic regression models were trained by using the *glm* method implemented in *caret* [42], a popular R [43] package. The scores were preprocessed using the *preProcess* function in *caret* for scaling and centering the data. The results of 4-fold cross-validation showed that the standard deviations of AUC ranged from 0.002 to 0.019 (Additional file 1: Table S3, Figure S1). Since some of the score sets are nested (i.e. some of the score set for a model is in a part of the score set in other models), ANOVA p-values were computed using the anova() function in R with the Chi square test to compare models that have nested relationships with the model using score Set 1. All ANOVA results indicated that using multiple and combined scores improved the performance of the model by showing that the reduction in the deviance is statistically significant.

The Logistic function is expressed as following:

$$F\left( t \right) = \frac{1}{{1 + e^{ - t} }},$$

where $$t = \beta_{0} + \beta_{1} s_{1} + \cdots + \beta_{n} s_{n}$$

Here, *s* represents each score (S_d_, S_e_, S_eg_, S_tr_, S_trg_, S_ta_, or S_tag_), β represents each coefficient, and *n* represents the number of scores used to construct a model (1 through 7 scores) depending on the score set that the model used.

## Additional file

**Additional file 1.** **Table S1**. Average structural similarity scores for the DDI/non–DDI pairs in the network of each De. **Table S2-1**. Top 10 predicted drugs with DDIs for warfarin. **Table S2-2**. Top 10 predicted drugs with DDIs for simvastatin. **Table S3**. Four-fold cross-validation test results. **Text S1**. Drugs that show DDI (DrugBank ID). **Figure S1**. Illustration of construction of training and test set for 4-fold cross validation. **Figure S2**. ROC curves using the models with score set 1 in a 4-fold validation.

## Abbreviations

DDI: drug–drug interaction
PK: pharmacokinetics
PD: pharmacodynamics
ADME: absorption, distribution, metabolism, and excretion
CYP: cytochrome
PBPK: physiologically based pharmacokinetic
QSAR: quantitative structure–activity relationship
Dq: query drug
De: drug to be examined

## References

[1] Health, United States, 2014 (5/2015)—hus14.pdf. http://www.cdc.gov/nchs/data/hus/hus14.pdf. Accessed 19 Sep 2016

[2] Research C for DE and drug interactions and labeling—preventable adverse drug reactions: a focus on drug interactions. http://www.fda.gov/Drugs/DevelopmentApprovalProcess/DevelopmentResources/DrugInteractionsLabeling/ucm110632.htm#Types%20of%20Drug%20Interactions. Accessed 13 Apr 2016

[3] Onakpoya IJ, Heneghan CJ, Aronson JK (2016). Post-marketing withdrawal of 462 medicinal products because of adverse drug reactions: a systematic review of the world literature. BMC Med.

[4] Palleria C, Di Paolo A, Giofrè C (2013). Pharmacokinetic drug–drug interaction and their implication in clinical management. J Res Med Sci.

[5] Ai N, Fan X, Ekins S (2015). In silico methods for predicting drug–drug interactions with cytochrome P-450s, transporters and beyond. Adv Drug Deliv Rev.

[6] Hinder M, Vogel HG, Maas J, Gebauer A (2011). Pharmacodynamic drug–drug interactions. Drug discovery and evaluation: methods in clinical pharmacology.

[7] Yamazaki S, Johnson TR, Smith BJ (2015). Prediction of drug–drug interactions with Crizotinib as the CYP3A substrate using a physiologically based pharmacokinetic model. Drug Metab Dispos Biol Fate Chem.

[8] Vilar S, Harpaz R, Uriarte E (2012). Drug–drug interaction through molecular structure similarity analysis. J Am Med Inform Assoc JAMIA.

[9] Vilar S, Uriarte E, Santana L (2014). Similarity-based modeling in large-scale prediction of drug–drug interactions. Nat Protoc.

[10] Gottlieb A, Stein GY, Oron Y (2012). INDI: a computational framework for inferring drug interactions and their associated recommendations. Mol Syst Biol.

[11] Vilar S, Uriarte E, Santana L (2014). State of the art and development of a drug–drug interaction large scale predictor based on 3D pharmacophoric similarity. Curr Drug Metab.

[12] Luo H, Zhang P, Huang H (2014). DDI-CPI, a server that predicts drug-drug interactions through implementing the chemical-protein interactome. Nucleic Acids Res.

[13] Cheng F, Zhao Z (2014). Machine learning-based prediction of drug–drug interactions by integrating drug phenotypic, therapeutic, chemical, and genomic properties. J Am Med Inform Assoc.

[14] Zakharov AV, Varlamova EV, Lagunin AA (2016). QSAR modeling and prediction of drug–drug interactions. Mol Pharm.

[15] Herrero-Zazo M, Segura-Bedmar I, Hastings J, Martínez P (2015). DINTO: using OWL ontologies and SWRL rules to infer drug–drug interactions and their mechanisms. J Chem Inf Model.

[16] Huang H, Zhang P, Qu XA (2014). Systematic prediction of drug combinations based on clinical side-effects. Sci Rep.

[17] Cami A, Manzi S, Arnold A, Reis BY (2013). Pharmacointeraction network models predict unknown drug–drug interactions. PLoS ONE.

[18] Zhang P, Wang F, Hu J, Sorrentino R (2015). Label propagation prediction of drug–drug interactions based on clinical side effects. Sci Rep.

[19] Iyer SV, Harpaz R, LePendu P (2014). Mining clinical text for signals of adverse drug–drug interactions. J Am Med Inform Assoc.

[20] Banda JM, Callahan A, Winnenburg R (2015). Feasibility of prioritizing drug–drug-event associations found in electronic health records. Drug Saf.

[21] Zhang L, Zhang Y, Huang S-M (2009). Scientific and regulatory perspectives on metabolizing enzyme–transporter interplay and its role in drug interactions: challenges in predicting drug interactions. Mol Pharm.

[22] van Waterschoot RAB, Schinkel AH (2011). A Critical analysis of the interplay between Cytochrome P450 3A and P-glycoprotein: recent insights from knockout and transgenic mice. Pharmacol Rev.

[23] WHO Model List of Essential Medicine. http://www.who.int/selection_medicines/committees/expert/20/EML_2015_FINAL_amended_AUG2015.pdf?ua=1. Accessed 28 April 2016

[24] Drug Interactions results—MICROMEDEX^®^. http://www.micromedexsolutions.com/micromedex2/librarian/PFDefaultActionId/evidencexpert.ShowDrugInteractionsResults. Accessed 9 May 2016

[25] Quercetin Drug Interactions—Drugs.com. http://www.drugs.com/drug-interactions/bioflavonoids,quercetin.html. Accessed 4 May 2016

[26] Di Bari L, Ripoli S, Pradhan S, Salvadori P (2010). Interactions between quercetin and warfarin for albumin binding: a new eye on food/drug interference. Chirality.

[27] Mahesha HG, Singh SA, Srinivasan N, Rao AGA (2006). A spectroscopic study of the interaction of isoflavones with human serum albumin. FEBS J.

[28] Dronabinol: MedlinePlus Drug Information. https:https://www.nlm.nih.gov/medlineplus/druginfo/meds/a607054.html. Accessed 3 May 2016

[29] Lovastatin and simvastatin/sitagliptin Drug Interactions—Drugs.com. http://www.drugs.com/drug-interactions/lovastatin-with-simvastatin-sitagliptin-1492-0-3347-0.html?professional=1. Accessed 4 May 2016

[30] Prednisolone and simvastatin/sitagliptin Drug Interactions—Drugs.com. http://www.drugs.com/drug-interactions/prednisolone-with-simvastatin-sitagliptin-1933-0-3347-0.html?professional=1. Accessed 4 May 2016

[31] Dexamethasone and simvastatin/sitagliptin Drug Interactions—Drugs.com. http://www.drugs.com/drug-interactions/dexamethasone-with-simvastatin-sitagliptin-810-0-3347-0.html?professional=1. Accessed 4 May 2016

[32] Prednisone and simvastatin/sitagliptin Drug Interactions—Drugs.com. http://www.drugs.com/drug-interactions/prednisone-with-simvastatin-sitagliptin-1936-0-3347-0.html?professional=1. Accessed 4 May 2016

[33] Simvastatin/sitagliptin and tacrolimus Drug Interactions—Drugs.com. http://www.drugs.com/drug-interactions/simvastatin-sitagliptin-with-tacrolimus-3347-0-2142-0.html?professional=1. Accessed 4 May 2016

[34] Sakaeda T, Takara K, Kakumoto M (2002). Simvastatin and lovastatin, but not pravastatin, interact with MDR1. J Pharm Pharmacol.

[35] Krysiak R, Kowalcze K, Bednarska-Czerwińska A, Okopień B (2016). The effect of simvastatin on plasma steroid hormone levels in wetformin-treated women with non-classic congenital adrenal hyperplasia. Exp Clin Endocrinol Diabetes.

[36] Lai Y, Hsiao P (2014). Beyond the ITC white paper: emerging sciences in drug transporters and opportunities for drug development. Curr Pharm Des.

[37] Wishart DS, Knox C, Guo AC (2006). DrugBank: a comprehensive resource for in silico drug discovery and exploration. Nucleic Acids Res.

[38] Whirl-Carrillo M, McDonagh EM, Hebert JM (2012). Pharmacogenomics knowledge for personalized medicine. Clin Pharmacol Ther.

[39] Stark C, Breitkreutz B-J, Reguly T (2006). BioGRID: a general repository for interaction datasets. Nucleic Acids Res.

[40] Hansen NT, Brunak S, Altman RB (2009). Generating genome-scale candidate gene lists for pharmacogenomics. Clin Pharmacol Ther.

[41] Kim S, Thiessen PA, Bolton EE (2016). PubChem substance and compound databases. Nucleic Acids Res.

[42] M Kuhn (2015) Contributions from J. Wing, S. Weston, A. Williams, C. Keefer, A. Engelhardt, T. Cooper, Z. Mayer, B. Kenkel, the R Core Team, M. Benesty, R. Lescarbeau, A. Ziem and L. Scrucca. caret: Classification and regression training. R package version 6.0-41

[43] R Core Team (2013) R: A language and environment for statistical computing. R foundation for statistical computing, Vienna, Austria. http://www.R-project.org/

