# Supplementary figures and images for: Predicting drug–drug interactions through drug structural similarities and interaction networks incorporating pharmacokinetics and pharmacodynamics knowledge
Source: J Cheminform. 2017 Mar 7;9:16. doi: 10.1186/s13321-017-0200-8 (PMC5340788; doi:10.1186/s13321-017-0200-8)

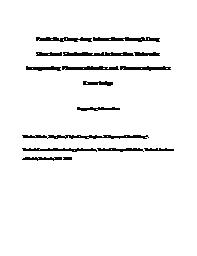

Supplement: Supplementary file 1 — Additional file 1. Table S1. Average structural similarity scores for the DDI/non–DDI pairs in the network of each De. Table S2-1. Top 10 predicted drugs with DDIs for warfarin. Table S2-2. Top 10 predicted drugs with DDIs for simvastatin. Table S3. Four-fold cross-validation test results. Text S1. Drugs that show DDI (DrugBank ID). Figure S1. Illustration of construction of training and test set for 4-fold cross validation. Figure S2. ROC curves using the models with score set 1 in a 4-fold validation. [file 13321_2017_200_MOESM1_ESM.xps › docProps/thumbnail.jpeg]

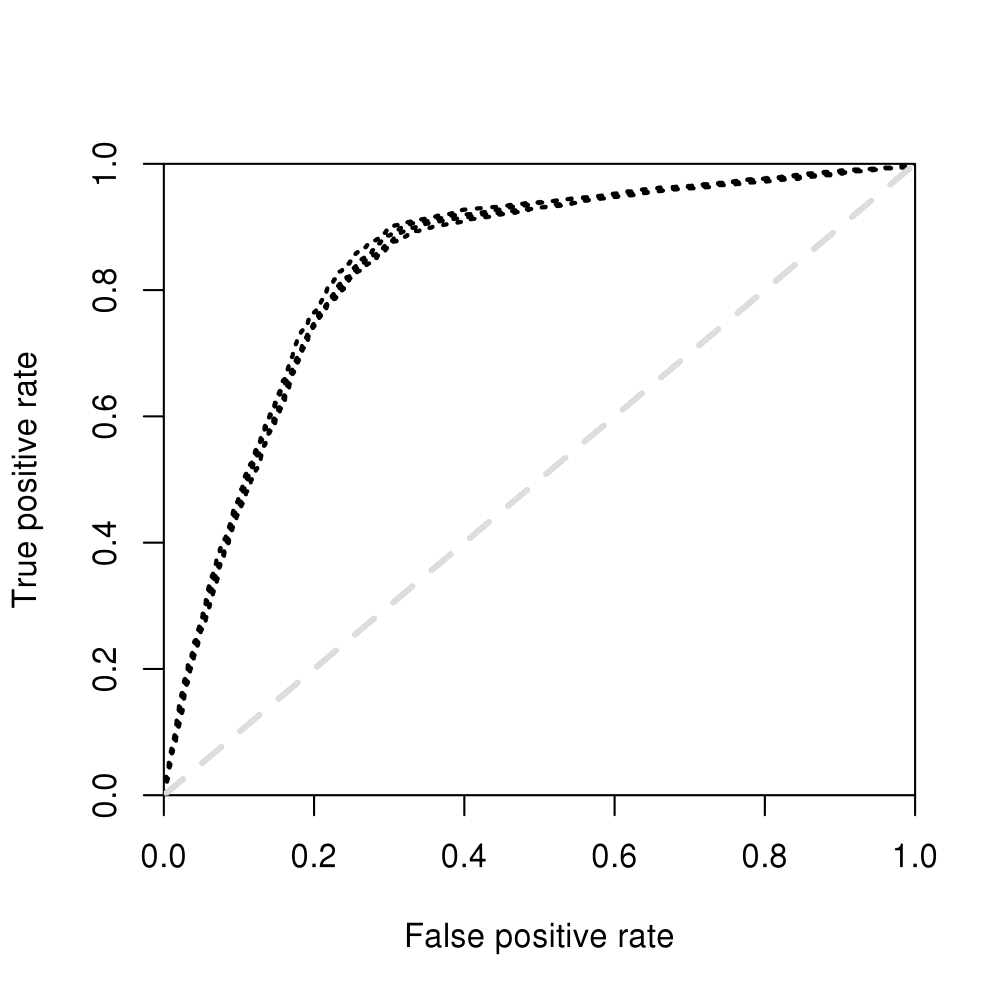

Supplement: Supplementary file 1 — Additional file 1. Table S1. Average structural similarity scores for the DDI/non–DDI pairs in the network of each De. Table S2-1. Top 10 predicted drugs with DDIs for warfarin. Table S2-2. Top 10 predicted drugs with DDIs for simvastatin. Table S3. Four-fold cross-validation test results. Text S1. Drugs that show DDI (DrugBank ID). Figure S1. Illustration of construction of training and test set for 4-fold cross validation. Figure S2. ROC curves using the models with score set 1 in a 4-fold validation. [file 13321_2017_200_MOESM1_ESM.xps › Resources/Images/image_43.png]

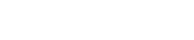

Supplement: Supplementary file 1 — Additional file 1. Table S1. Average structural similarity scores for the DDI/non–DDI pairs in the network of each De. Table S2-1. Top 10 predicted drugs with DDIs for warfarin. Table S2-2. Top 10 predicted drugs with DDIs for simvastatin. Table S3. Four-fold cross-validation test results. Text S1. Drugs that show DDI (DrugBank ID). Figure S1. Illustration of construction of training and test set for 4-fold cross validation. Figure S2. ROC curves using the models with score set 1 in a 4-fold validation. [file 13321_2017_200_MOESM1_ESM.xps › Resources/Images/image_42.png]

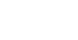

Supplement: Supplementary file 1 — Additional file 1. Table S1. Average structural similarity scores for the DDI/non–DDI pairs in the network of each De. Table S2-1. Top 10 predicted drugs with DDIs for warfarin. Table S2-2. Top 10 predicted drugs with DDIs for simvastatin. Table S3. Four-fold cross-validation test results. Text S1. Drugs that show DDI (DrugBank ID). Figure S1. Illustration of construction of training and test set for 4-fold cross validation. Figure S2. ROC curves using the models with score set 1 in a 4-fold validation. [file 13321_2017_200_MOESM1_ESM.xps › Resources/Images/image_35.png]

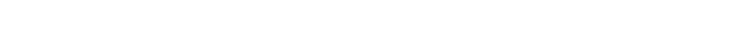

Supplement: Supplementary file 1 — Additional file 1. Table S1. Average structural similarity scores for the DDI/non–DDI pairs in the network of each De. Table S2-1. Top 10 predicted drugs with DDIs for warfarin. Table S2-2. Top 10 predicted drugs with DDIs for simvastatin. Table S3. Four-fold cross-validation test results. Text S1. Drugs that show DDI (DrugBank ID). Figure S1. Illustration of construction of training and test set for 4-fold cross validation. Figure S2. ROC curves using the models with score set 1 in a 4-fold validation. [file 13321_2017_200_MOESM1_ESM.xps › Resources/Images/image_38.png]

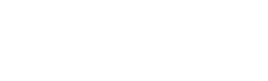

Supplement: Supplementary file 1 — Additional file 1. Table S1. Average structural similarity scores for the DDI/non–DDI pairs in the network of each De. Table S2-1. Top 10 predicted drugs with DDIs for warfarin. Table S2-2. Top 10 predicted drugs with DDIs for simvastatin. Table S3. Four-fold cross-validation test results. Text S1. Drugs that show DDI (DrugBank ID). Figure S1. Illustration of construction of training and test set for 4-fold cross validation. Figure S2. ROC curves using the models with score set 1 in a 4-fold validation. [file 13321_2017_200_MOESM1_ESM.xps › Resources/Images/image_32.png]

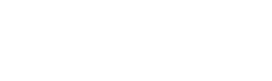

Supplement: Supplementary file 1 — Additional file 1. Table S1. Average structural similarity scores for the DDI/non–DDI pairs in the network of each De. Table S2-1. Top 10 predicted drugs with DDIs for warfarin. Table S2-2. Top 10 predicted drugs with DDIs for simvastatin. Table S3. Four-fold cross-validation test results. Text S1. Drugs that show DDI (DrugBank ID). Figure S1. Illustration of construction of training and test set for 4-fold cross validation. Figure S2. ROC curves using the models with score set 1 in a 4-fold validation. [file 13321_2017_200_MOESM1_ESM.xps › Resources/Images/image_31.png]

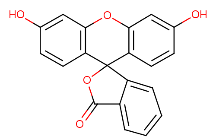

Supplement: Supplementary file 1 — Additional file 1. Table S1. Average structural similarity scores for the DDI/non–DDI pairs in the network of each De. Table S2-1. Top 10 predicted drugs with DDIs for warfarin. Table S2-2. Top 10 predicted drugs with DDIs for simvastatin. Table S3. Four-fold cross-validation test results. Text S1. Drugs that show DDI (DrugBank ID). Figure S1. Illustration of construction of training and test set for 4-fold cross validation. Figure S2. ROC curves using the models with score set 1 in a 4-fold validation. [file 13321_2017_200_MOESM1_ESM.xps › Resources/Images/image_7.png]

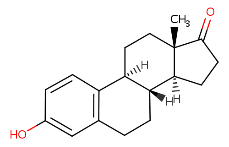

Supplement: Supplementary file 1 — Additional file 1. Table S1. Average structural similarity scores for the DDI/non–DDI pairs in the network of each De. Table S2-1. Top 10 predicted drugs with DDIs for warfarin. Table S2-2. Top 10 predicted drugs with DDIs for simvastatin. Table S3. Four-fold cross-validation test results. Text S1. Drugs that show DDI (DrugBank ID). Figure S1. Illustration of construction of training and test set for 4-fold cross validation. Figure S2. ROC curves using the models with score set 1 in a 4-fold validation. [file 13321_2017_200_MOESM1_ESM.xps › Resources/Images/image_8.png]

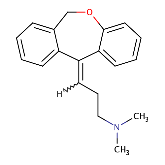

Supplement: Supplementary file 1 — Additional file 1. Table S1. Average structural similarity scores for the DDI/non–DDI pairs in the network of each De. Table S2-1. Top 10 predicted drugs with DDIs for warfarin. Table S2-2. Top 10 predicted drugs with DDIs for simvastatin. Table S3. Four-fold cross-validation test results. Text S1. Drugs that show DDI (DrugBank ID). Figure S1. Illustration of construction of training and test set for 4-fold cross validation. Figure S2. ROC curves using the models with score set 1 in a 4-fold validation. [file 13321_2017_200_MOESM1_ESM.xps › Resources/Images/image_9.png]

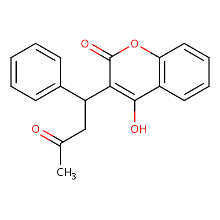

Supplement: Supplementary file 1 — Additional file 1. Table S1. Average structural similarity scores for the DDI/non–DDI pairs in the network of each De. Table S2-1. Top 10 predicted drugs with DDIs for warfarin. Table S2-2. Top 10 predicted drugs with DDIs for simvastatin. Table S3. Four-fold cross-validation test results. Text S1. Drugs that show DDI (DrugBank ID). Figure S1. Illustration of construction of training and test set for 4-fold cross validation. Figure S2. ROC curves using the models with score set 1 in a 4-fold validation. [file 13321_2017_200_MOESM1_ESM.xps › Resources/Images/image_10.png]

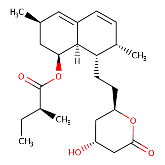

Supplement: Supplementary file 1 — Additional file 1. Table S1. Average structural similarity scores for the DDI/non–DDI pairs in the network of each De. Table S2-1. Top 10 predicted drugs with DDIs for warfarin. Table S2-2. Top 10 predicted drugs with DDIs for simvastatin. Table S3. Four-fold cross-validation test results. Text S1. Drugs that show DDI (DrugBank ID). Figure S1. Illustration of construction of training and test set for 4-fold cross validation. Figure S2. ROC curves using the models with score set 1 in a 4-fold validation. [file 13321_2017_200_MOESM1_ESM.xps › Resources/Images/image_11.png]

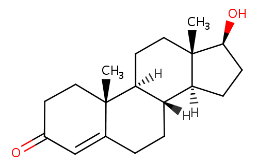

Supplement: Supplementary file 1 — Additional file 1. Table S1. Average structural similarity scores for the DDI/non–DDI pairs in the network of each De. Table S2-1. Top 10 predicted drugs with DDIs for warfarin. Table S2-2. Top 10 predicted drugs with DDIs for simvastatin. Table S3. Four-fold cross-validation test results. Text S1. Drugs that show DDI (DrugBank ID). Figure S1. Illustration of construction of training and test set for 4-fold cross validation. Figure S2. ROC curves using the models with score set 1 in a 4-fold validation. [file 13321_2017_200_MOESM1_ESM.xps › Resources/Images/image_12.png]

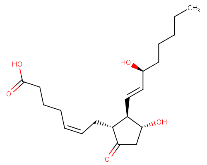

Supplement: Supplementary file 1 — Additional file 1. Table S1. Average structural similarity scores for the DDI/non–DDI pairs in the network of each De. Table S2-1. Top 10 predicted drugs with DDIs for warfarin. Table S2-2. Top 10 predicted drugs with DDIs for simvastatin. Table S3. Four-fold cross-validation test results. Text S1. Drugs that show DDI (DrugBank ID). Figure S1. Illustration of construction of training and test set for 4-fold cross validation. Figure S2. ROC curves using the models with score set 1 in a 4-fold validation. [file 13321_2017_200_MOESM1_ESM.xps › Resources/Images/image_13.png]

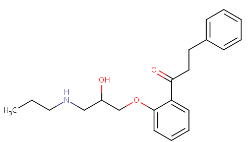

Supplement: Supplementary file 1 — Additional file 1. Table S1. Average structural similarity scores for the DDI/non–DDI pairs in the network of each De. Table S2-1. Top 10 predicted drugs with DDIs for warfarin. Table S2-2. Top 10 predicted drugs with DDIs for simvastatin. Table S3. Four-fold cross-validation test results. Text S1. Drugs that show DDI (DrugBank ID). Figure S1. Illustration of construction of training and test set for 4-fold cross validation. Figure S2. ROC curves using the models with score set 1 in a 4-fold validation. [file 13321_2017_200_MOESM1_ESM.xps › Resources/Images/image_6.png]

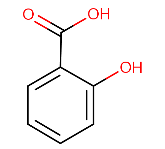

Supplement: Supplementary file 1 — Additional file 1. Table S1. Average structural similarity scores for the DDI/non–DDI pairs in the network of each De. Table S2-1. Top 10 predicted drugs with DDIs for warfarin. Table S2-2. Top 10 predicted drugs with DDIs for simvastatin. Table S3. Four-fold cross-validation test results. Text S1. Drugs that show DDI (DrugBank ID). Figure S1. Illustration of construction of training and test set for 4-fold cross validation. Figure S2. ROC curves using the models with score set 1 in a 4-fold validation. [file 13321_2017_200_MOESM1_ESM.xps › Resources/Images/image_5.png]

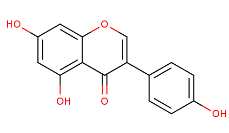

Supplement: Supplementary file 1 — Additional file 1. Table S1. Average structural similarity scores for the DDI/non–DDI pairs in the network of each De. Table S2-1. Top 10 predicted drugs with DDIs for warfarin. Table S2-2. Top 10 predicted drugs with DDIs for simvastatin. Table S3. Four-fold cross-validation test results. Text S1. Drugs that show DDI (DrugBank ID). Figure S1. Illustration of construction of training and test set for 4-fold cross validation. Figure S2. ROC curves using the models with score set 1 in a 4-fold validation. [file 13321_2017_200_MOESM1_ESM.xps › Resources/Images/image_4.png]

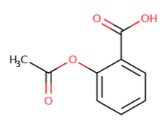

Supplement: Supplementary file 1 — Additional file 1. Table S1. Average structural similarity scores for the DDI/non–DDI pairs in the network of each De. Table S2-1. Top 10 predicted drugs with DDIs for warfarin. Table S2-2. Top 10 predicted drugs with DDIs for simvastatin. Table S3. Four-fold cross-validation test results. Text S1. Drugs that show DDI (DrugBank ID). Figure S1. Illustration of construction of training and test set for 4-fold cross validation. Figure S2. ROC curves using the models with score set 1 in a 4-fold validation. [file 13321_2017_200_MOESM1_ESM.xps › Resources/Images/image_0.png]

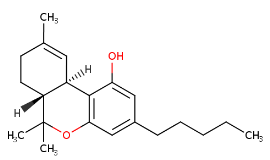

Supplement: Supplementary file 1 — Additional file 1. Table S1. Average structural similarity scores for the DDI/non–DDI pairs in the network of each De. Table S2-1. Top 10 predicted drugs with DDIs for warfarin. Table S2-2. Top 10 predicted drugs with DDIs for simvastatin. Table S3. Four-fold cross-validation test results. Text S1. Drugs that show DDI (DrugBank ID). Figure S1. Illustration of construction of training and test set for 4-fold cross validation. Figure S2. ROC curves using the models with score set 1 in a 4-fold validation. [file 13321_2017_200_MOESM1_ESM.xps › Resources/Images/image_1.png]

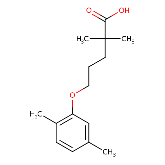

Supplement: Supplementary file 1 — Additional file 1. Table S1. Average structural similarity scores for the DDI/non–DDI pairs in the network of each De. Table S2-1. Top 10 predicted drugs with DDIs for warfarin. Table S2-2. Top 10 predicted drugs with DDIs for simvastatin. Table S3. Four-fold cross-validation test results. Text S1. Drugs that show DDI (DrugBank ID). Figure S1. Illustration of construction of training and test set for 4-fold cross validation. Figure S2. ROC curves using the models with score set 1 in a 4-fold validation. [file 13321_2017_200_MOESM1_ESM.xps › Resources/Images/image_2.png]

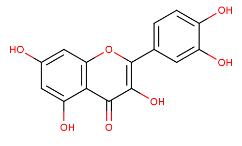

Supplement: Supplementary file 1 — Additional file 1. Table S1. Average structural similarity scores for the DDI/non–DDI pairs in the network of each De. Table S2-1. Top 10 predicted drugs with DDIs for warfarin. Table S2-2. Top 10 predicted drugs with DDIs for simvastatin. Table S3. Four-fold cross-validation test results. Text S1. Drugs that show DDI (DrugBank ID). Figure S1. Illustration of construction of training and test set for 4-fold cross validation. Figure S2. ROC curves using the models with score set 1 in a 4-fold validation. [file 13321_2017_200_MOESM1_ESM.xps › Resources/Images/image_3.png]

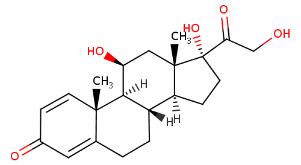

Supplement: Supplementary file 1 — Additional file 1. Table S1. Average structural similarity scores for the DDI/non–DDI pairs in the network of each De. Table S2-1. Top 10 predicted drugs with DDIs for warfarin. Table S2-2. Top 10 predicted drugs with DDIs for simvastatin. Table S3. Four-fold cross-validation test results. Text S1. Drugs that show DDI (DrugBank ID). Figure S1. Illustration of construction of training and test set for 4-fold cross validation. Figure S2. ROC curves using the models with score set 1 in a 4-fold validation. [file 13321_2017_200_MOESM1_ESM.xps › Resources/Images/image_14.png]

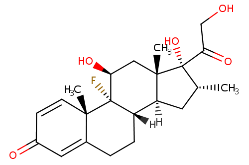

Supplement: Supplementary file 1 — Additional file 1. Table S1. Average structural similarity scores for the DDI/non–DDI pairs in the network of each De. Table S2-1. Top 10 predicted drugs with DDIs for warfarin. Table S2-2. Top 10 predicted drugs with DDIs for simvastatin. Table S3. Four-fold cross-validation test results. Text S1. Drugs that show DDI (DrugBank ID). Figure S1. Illustration of construction of training and test set for 4-fold cross validation. Figure S2. ROC curves using the models with score set 1 in a 4-fold validation. [file 13321_2017_200_MOESM1_ESM.xps › Resources/Images/image_15.png]

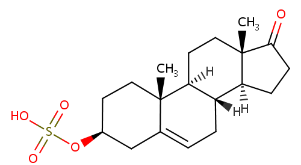

Supplement: Supplementary file 1 — Additional file 1. Table S1. Average structural similarity scores for the DDI/non–DDI pairs in the network of each De. Table S2-1. Top 10 predicted drugs with DDIs for warfarin. Table S2-2. Top 10 predicted drugs with DDIs for simvastatin. Table S3. Four-fold cross-validation test results. Text S1. Drugs that show DDI (DrugBank ID). Figure S1. Illustration of construction of training and test set for 4-fold cross validation. Figure S2. ROC curves using the models with score set 1 in a 4-fold validation. [file 13321_2017_200_MOESM1_ESM.xps › Resources/Images/image_16.png]

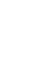

Supplement: Supplementary file 1 — Additional file 1. Table S1. Average structural similarity scores for the DDI/non–DDI pairs in the network of each De. Table S2-1. Top 10 predicted drugs with DDIs for warfarin. Table S2-2. Top 10 predicted drugs with DDIs for simvastatin. Table S3. Four-fold cross-validation test results. Text S1. Drugs that show DDI (DrugBank ID). Figure S1. Illustration of construction of training and test set for 4-fold cross validation. Figure S2. ROC curves using the models with score set 1 in a 4-fold validation. [file 13321_2017_200_MOESM1_ESM.xps › Resources/Images/image_23.png]

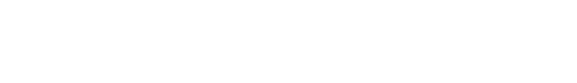

Supplement: Supplementary file 1 — Additional file 1. Table S1. Average structural similarity scores for the DDI/non–DDI pairs in the network of each De. Table S2-1. Top 10 predicted drugs with DDIs for warfarin. Table S2-2. Top 10 predicted drugs with DDIs for simvastatin. Table S3. Four-fold cross-validation test results. Text S1. Drugs that show DDI (DrugBank ID). Figure S1. Illustration of construction of training and test set for 4-fold cross validation. Figure S2. ROC curves using the models with score set 1 in a 4-fold validation. [file 13321_2017_200_MOESM1_ESM.xps › Resources/Images/image_26.png]

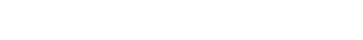

Supplement: Supplementary file 1 — Additional file 1. Table S1. Average structural similarity scores for the DDI/non–DDI pairs in the network of each De. Table S2-1. Top 10 predicted drugs with DDIs for warfarin. Table S2-2. Top 10 predicted drugs with DDIs for simvastatin. Table S3. Four-fold cross-validation test results. Text S1. Drugs that show DDI (DrugBank ID). Figure S1. Illustration of construction of training and test set for 4-fold cross validation. Figure S2. ROC curves using the models with score set 1 in a 4-fold validation. [file 13321_2017_200_MOESM1_ESM.xps › Resources/Images/image_27.png]

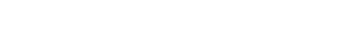

Supplement: Supplementary file 1 — Additional file 1. Table S1. Average structural similarity scores for the DDI/non–DDI pairs in the network of each De. Table S2-1. Top 10 predicted drugs with DDIs for warfarin. Table S2-2. Top 10 predicted drugs with DDIs for simvastatin. Table S3. Four-fold cross-validation test results. Text S1. Drugs that show DDI (DrugBank ID). Figure S1. Illustration of construction of training and test set for 4-fold cross validation. Figure S2. ROC curves using the models with score set 1 in a 4-fold validation. [file 13321_2017_200_MOESM1_ESM.xps › Resources/Images/image_28.png]

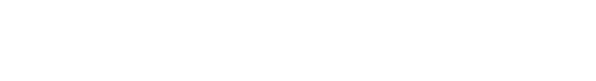

Supplement: Supplementary file 1 — Additional file 1. Table S1. Average structural similarity scores for the DDI/non–DDI pairs in the network of each De. Table S2-1. Top 10 predicted drugs with DDIs for warfarin. Table S2-2. Top 10 predicted drugs with DDIs for simvastatin. Table S3. Four-fold cross-validation test results. Text S1. Drugs that show DDI (DrugBank ID). Figure S1. Illustration of construction of training and test set for 4-fold cross validation. Figure S2. ROC curves using the models with score set 1 in a 4-fold validation. [file 13321_2017_200_MOESM1_ESM.xps › Resources/Images/image_29.png]

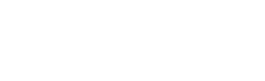

Supplement: Supplementary file 1 — Additional file 1. Table S1. Average structural similarity scores for the DDI/non–DDI pairs in the network of each De. Table S2-1. Top 10 predicted drugs with DDIs for warfarin. Table S2-2. Top 10 predicted drugs with DDIs for simvastatin. Table S3. Four-fold cross-validation test results. Text S1. Drugs that show DDI (DrugBank ID). Figure S1. Illustration of construction of training and test set for 4-fold cross validation. Figure S2. ROC curves using the models with score set 1 in a 4-fold validation. [file 13321_2017_200_MOESM1_ESM.xps › Resources/Images/image_30.png]

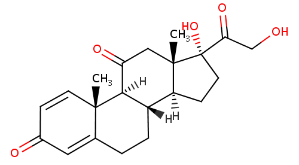

Supplement: Supplementary file 1 — Additional file 1. Table S1. Average structural similarity scores for the DDI/non–DDI pairs in the network of each De. Table S2-1. Top 10 predicted drugs with DDIs for warfarin. Table S2-2. Top 10 predicted drugs with DDIs for simvastatin. Table S3. Four-fold cross-validation test results. Text S1. Drugs that show DDI (DrugBank ID). Figure S1. Illustration of construction of training and test set for 4-fold cross validation. Figure S2. ROC curves using the models with score set 1 in a 4-fold validation. [file 13321_2017_200_MOESM1_ESM.xps › Resources/Images/image_17.png]

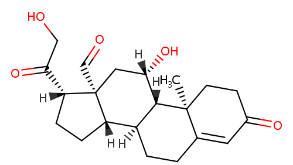

Supplement: Supplementary file 1 — Additional file 1. Table S1. Average structural similarity scores for the DDI/non–DDI pairs in the network of each De. Table S2-1. Top 10 predicted drugs with DDIs for warfarin. Table S2-2. Top 10 predicted drugs with DDIs for simvastatin. Table S3. Four-fold cross-validation test results. Text S1. Drugs that show DDI (DrugBank ID). Figure S1. Illustration of construction of training and test set for 4-fold cross validation. Figure S2. ROC curves using the models with score set 1 in a 4-fold validation. [file 13321_2017_200_MOESM1_ESM.xps › Resources/Images/image_18.png]

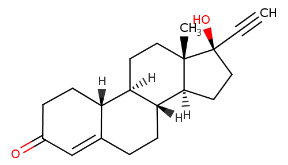

Supplement: Supplementary file 1 — Additional file 1. Table S1. Average structural similarity scores for the DDI/non–DDI pairs in the network of each De. Table S2-1. Top 10 predicted drugs with DDIs for warfarin. Table S2-2. Top 10 predicted drugs with DDIs for simvastatin. Table S3. Four-fold cross-validation test results. Text S1. Drugs that show DDI (DrugBank ID). Figure S1. Illustration of construction of training and test set for 4-fold cross validation. Figure S2. ROC curves using the models with score set 1 in a 4-fold validation. [file 13321_2017_200_MOESM1_ESM.xps › Resources/Images/image_19.png]

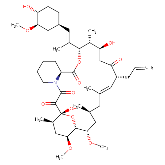

Supplement: Supplementary file 1 — Additional file 1. Table S1. Average structural similarity scores for the DDI/non–DDI pairs in the network of each De. Table S2-1. Top 10 predicted drugs with DDIs for warfarin. Table S2-2. Top 10 predicted drugs with DDIs for simvastatin. Table S3. Four-fold cross-validation test results. Text S1. Drugs that show DDI (DrugBank ID). Figure S1. Illustration of construction of training and test set for 4-fold cross validation. Figure S2. ROC curves using the models with score set 1 in a 4-fold validation. [file 13321_2017_200_MOESM1_ESM.xps › Resources/Images/image_20.png]

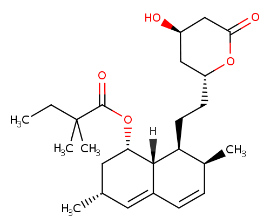

Supplement: Supplementary file 1 — Additional file 1. Table S1. Average structural similarity scores for the DDI/non–DDI pairs in the network of each De. Table S2-1. Top 10 predicted drugs with DDIs for warfarin. Table S2-2. Top 10 predicted drugs with DDIs for simvastatin. Table S3. Four-fold cross-validation test results. Text S1. Drugs that show DDI (DrugBank ID). Figure S1. Illustration of construction of training and test set for 4-fold cross validation. Figure S2. ROC curves using the models with score set 1 in a 4-fold validation. [file 13321_2017_200_MOESM1_ESM.xps › Resources/Images/image_21.png]

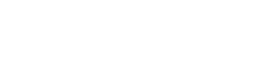

Supplement: Supplementary file 1 — Additional file 1. Table S1. Average structural similarity scores for the DDI/non–DDI pairs in the network of each De. Table S2-1. Top 10 predicted drugs with DDIs for warfarin. Table S2-2. Top 10 predicted drugs with DDIs for simvastatin. Table S3. Four-fold cross-validation test results. Text S1. Drugs that show DDI (DrugBank ID). Figure S1. Illustration of construction of training and test set for 4-fold cross validation. Figure S2. ROC curves using the models with score set 1 in a 4-fold validation. [file 13321_2017_200_MOESM1_ESM.xps › Resources/Images/image_33.png]
